# Dental Caries Experience in Older Adults With Parkinson's Disease: A Systematic Review and Meta‐Analysis

**DOI:** 10.1111/scd.70230

**Published:** 2026-08-10

**Authors:** Victor Zanetti Drumond, Thaís Alves de Oliveira, José Alcides Almeida de Arruda, Raissa Costa Leal Pavione, Najara Barbosa Rocha, Ricardo Alves Mesquita, Lucas Guimarães Abreu

**Affiliations:** ^1^ Department of Oral Surgery, Pathology, and Clinical Dentistry, School of Dentistry Universidade Federal de Minas Gerais Belo Horizonte Brazil; ^2^ School of Dentistry Universidade Federal de Minas Gerais Belo Horizonte Brazil; ^3^ School of Dentistry Universidade Federal de Alfenas Alfenas Brazil; ^4^ Private practice, Ipatinga Minas Gerais Brazil; ^5^ Department of Social and Preventive Dentistry, School of Dentistry Universidade Federal de Minas Gerais Belo Horizonte Brazil; ^6^ Department of Child and Adolescent Oral Health, School of Dentistry Universidade Federal de Minas Gerais Belo Horizonte Brazil

**Keywords:** dental caries, meta‐analysis, neurodegenerative disorder, Parkinson's disease, tooth decay

## Abstract

**Aims:**

This systematic review and meta‐analysis assessed whether individuals with Parkinson's disease (PD) have higher dental caries rates than those without PD.

**Methods:**

Seven electronic databases and gray literature sources were systematically searched for observational studies comparing dental caries between individuals with and without PD. Risk of bias was assessed using the Newcastle‐Ottawa Scale. Meta‐analyses of continuous data were performed, reporting mean differences (MD) with 95% confidence intervals (CI). The strength of evidence was appraised using the GRADE framework.

**Results:**

Of 389 retrieved records, 10 studies were included, comprising 1726 individuals (573 with PD; 1153 controls). All studies employed a cross‐sectional design with a control group and exhibited a moderate risk of bias. Meta‐analyses revealed no significant differences in the decayed, missing, and filled teeth (DMFT) index (*k* = 5) (MD = −0.30; 95% CI = −5.18–4.57) or untreated dental caries (*k* = 4) (MD = 0.73; 95% CI = −1.66–3.12). The strength of evidence was classified as low.

**Conclusion:**

To date, very‐low‐certainty evidence does not demonstrate a statistically significant difference in dental caries experience between older adults with and without PD. These findings should be interpreted cautiously and should not be taken as evidence that PD is unrelated to current or future dental caries susceptibility.

## 1 Introduction

Parkinson's disease (PD) is a complex, age‐related neurodegenerative disorder that significantly impairs motor performance, profoundly affecting patients' autonomy and quality of life [1]. The term PD originates from the seminal work of James Parkinson, a British polymath and surgeon, who, in 1817, published *An Essay on the Shaking Palsy*, the first detailed description of *paralysis agitans*. Later, the French neurologist Jean‐Martin Charcot formally coined the term “PD”, refining its clinical characterization [2, 3]. PD predominantly affects individuals aged 65 to 70 years, with a higher prevalence in men. Its etiology is multifactorial, involving genetic, epigenetic, and environmental factors, such as exposure to neurotoxic agents (e.g., agrochemicals and pesticides) [4, 5]. According to the Global Burden of Disease study, the global prevalence of PD reached approximately 6 million individuals in 2016 [6].

The pathophysiology of PD is primarily characterized by the progressive degeneration of dopaminergic neurons within the substantia nigra pars compacta, accompanied by the pathological deposition of *α*‐synuclein. These processes lead to hallmark motor symptoms, including bradykinesia, resting tremor, and rigidity [7, 8]. However, PD is increasingly recognized as a multisystem disorder, with non‐motor symptoms such as orthostatic hypotension, urinary dysfunction, sleep disturbances, and depressive symptoms being highly prevalent [9, 10]. Despite the expanding knowledge of PD's systemic manifestations, oral health has received relatively little attention in this context [11, 12].

Oral health is integral to overall well‐being and is particularly vulnerable in individuals with PD due to motor impairments that hinder routine oral hygiene practices, leading to an increased risk of oral diseases [11, 13]. Dental caries, one of the most prevalent noncommunicable diseases worldwide, is characterized by dysbiosis and the demineralization of tooth structures due to acidogenic bacterial metabolism, particularly *Streptococcus mutans* [14, 15, 16]. The pathogenesis of dental caries is strongly influenced by dietary habits, socioeconomic status, and oral hygiene behaviors [17, 18, 19], all of which may be further compromised by PD‐associated motor dysfunction and cognitive decline habits [11].

In recent years, multiple reviews have addressed the oral health status of patients with PD. Verhoeff et al. (2023) [12] systematically reviewed the oral health burden in PD, identifying a higher prevalence of dental biofilm, gingival bleeding, periodontal pockets ≥4 mm, tooth mobility, and dental caries in PD patients compared to controls. However, despite providing descriptive data, this review did not conduct a meta‐analysis. Tebbutt et al. (2024) [11] conducted a scoping review on oral health experiences in PD patients, highlighting challenges such as limited access to dental care, reduced oral hygiene behaviors, and an increased need for professional dental interventions. Despite its broad overview, that study did not include a quantitative synthesis or focus on dental caries specifically. Martimbianco et al. [20] focused on evidence‐based recommendations for oral health care in PD, emphasizing the prevalence of gingival recession, periodontal disease, dental calculus, tooth loss, and xerostomia; yet without quantitatively assessing dental caries risk. Chen et al. [17] and Suryawanshi et al. [21] conducted meta‐analyses focusing on periodontal health in PD, confirming a higher prevalence of periodontitis and periodontal attachment loss. Collectively, these previous reviews underscore the substantial burden of oral health deterioration in PD patients, yet none have specifically addressed dental caries with a meta‐analytic approach. This omission represents a critical gap in the literature, as dental caries prevalence and severity may differ from other oral diseases in PD due to distinct etiopathogenic mechanisms [18, 19, 22].

Given the impact of both PD and dental caries on quality of life, [23, 24, 25] a systematic analysis of their association is both timely and necessary. The purpose of the present systematic review and meta‐analysis was to investigate the link between PD and dental caries.

## 2 Methods

### 2.1 Protocol Registration and Reporting Guideline

This systematic review and meta‐analysis was performed in accordance with the Meta‐analysis of Observational Studies in Epidemiology (MOOSE) guidelines [26]. The study protocol was registered in PROSPERO (https://www.crd.york.ac.uk/prospero/) under the registration number CRD42022312874.

### 2.2 Eligibility Criteria

Eligible studies included observational studies, that is, case‐control, cross‐sectional with a control group, and cohort studies, comparing individuals with PD and non‐PD controls in relation to dental caries. Other study designs were excluded. No restrictions were applied regarding publication date or language. Eligibility was structured using the PECO framework: P (Population): individuals; E (Exposure): PD; C (Comparison): non‐PD controls; O (Outcome): dental caries.

### 2.3 Databases and Search Strategy

Electronic searches were conducted from database inception to January 2025 across the following databases: PubMed (National Library of Medicine), Web of Science (Clarivate Analytics), Scopus (Elsevier), Embase (Elsevier), CINAHL (EBSCO), Ovid (Wolters Kluwer), and PsycINFO (APA). An update on electronic search took place in June 2025. The search strategy combined terms for PD and dental caries using Boolean operators (“AND” and “OR”). An initial search strategy was designed for PubMed and adapted to comply with the syntax requirements of other databases (Supplementary File 1). Additional searches were conducted in OpenGrey and Google Scholar, with results restricted to the first 200 records. The reference lists of included studies were also examined to identify additional relevant articles.

### 2.4 Screening Procedure

Two independent and previously calibrated authors (V.Z.D. and T.A.O.) conducted the screening process in two distinct phases. Phase 1 involved screening titles and abstracts against the predefined inclusion criteria, with potentially relevant studies progressing to phase 2, where full texts were evaluated. Only studies that fully met the eligibility criteria were included. Discrepancies were resolved by a third author (J.A.A.A.). To ensure rigorous reference management and duplicate removal, records were processed using EndNote Web (Clarivate Analytics, London, UK). The kappa coefficient was calculated to assess inter‐reviewer agreement [27].

### 2.5 Data Gathering

Data collection process was conducted by two authors (V.Z.D. and T.A.O.), with one extracting the information and the other verifying its accuracy. The following variables were systematically recorded in a standardized table: (i) study characteristics: first author's surname, year of publication, study design, and country; (ii) sample characteristics: sample size, age distribution, and sex distribution, (iii) methodological aspects: exposure and outcome assessment methods; and (iv) quantitative data: effect measures assessing the exposure‐outcome association.

### 2.6 Risk of Bias Appraisal

The risk of bias in included studies was assessed using the Newcastle‐Ottawa Scale adapted for cross‐sectional studies. Although the eligibility criteria allowed case‐control, cross‐sectional, and cohort designs, all studies included after full‐text screening provided cross‐sectional comparative data with a control group; therefore, the cross‐sectional version of the tool was applied consistently. The tool evaluates three key domains: selection bias, including sample representativeness, sample size, response rate, and exposure assessment; comparability, including strategies used to control confounders; and outcome assessment, including quality of outcome measurement and statistical analyses employed [28].

### 2.7 Quantitative Synthesis

A random‐effects meta‐analysis was conducted to pool effect sizes from primary studies on the mean number of dental caries and the decayed, missing, and filled teeth (DMFT) index in permanent dentition. Results were expressed as mean difference (MD) with 95% confidence intervals (CI). Between‐study variance (*τ* ^2^) was estimated using restricted maximum likelihood [29]. The Hartung‐Knapp adjustment was applied to mitigate Type I and Type II errors [30]. Heterogeneity was assessed using the *Q‐*Statistic, and its magnitude was quantified using *I* ^2^, *τ*, and *τ* ^2^ [31]. When necessary, standard errors (SE) were converted to standard deviations (SD) by multiplying the SE by the square root of the sample size (N) [32]. Statistical analyses were conducted using R (R Foundation for Statistical Computing, Vienna, Austria R) with the *meta* package [33] within RStudio (RStudio, Boston, USA). To explore potential heterogeneity, Baujat plots were generated [34].

### 2.8 Strength of the Evidence

The overall strength of the evidence was appraised using the Grading of Recommendations, Assessment, Development, and Evaluation (GRADE) approach [35].

## 3 Results

### 3.1 Search Results and Screening Procedure

**FIGURE 1:**
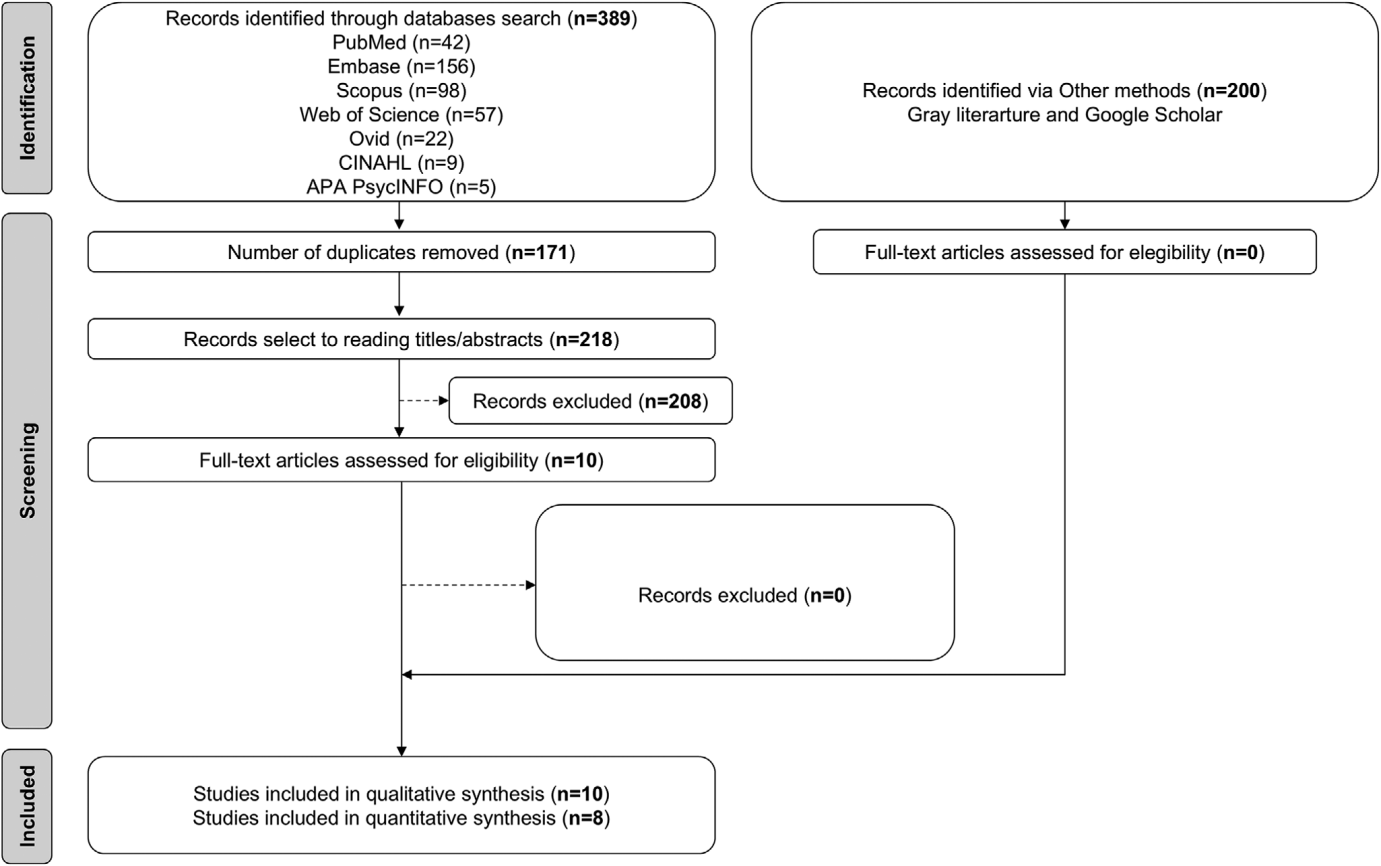
Flowchart depicting the screening procedure.

The electronic searches retrieved 389 records, of which 218 studies remained after duplicate removal. After screening titles and abstracts, 10 studies were deemed eligible for full‐text review. All 10 records met the eligibility criteria and were included in the qualitative synthesis. The screening procedure is illustrated in Figure 1.

### 3.2 General Characteristics of the Included Studies

All 10 included studies employed a cross‐sectional design with a control group [36, 37, 38, 39, 40, 41, 42, 43, 44, 45]. The publication period ranged from 1992 [43] to 2022 [40]. The studies were conducted in Germany (*n* = 2), Japan (*n* = 2), Brazil (*n* = 1), Italy (*n* = 1), Iceland (*n* = 1), the Netherlands (*n* = 1), Spain (*n* = 1), and Sweden (*n* = 1). The studies collectively evaluated 1726 individuals, comprising 573 individuals with PD and 1153 controls. Participant ages ranged from 60 [38] to 78 years [37], with mean ages of 68.4 years in the PD group and 66.2 years in the control group. Study settings included university dental clinics (*n* = 3), specialized centers (*n* = 2), university hospitals (*n* = 1), general hospitals (*n* = 2), and university neurology clinics (*n* = 1). The study setting was not reported in one [41] instance (Supplementary File 2).

### 3.3 Summary of Findings from the Included Studies

#### 3.3.1 Exposure Appraisal

In five studies [37, 39, 41, 42, 44], PD severity and progression were assessed using a validated scale, specifically the Hoehn and Yahr scale. In one study [43], the criteria for PD assessment were not reported. In four studies [36, 38, 40, 45], information on PD severity was unavailable.

#### 3.3.2 Outcome Appraisal

Dental caries was assessed through clinical examination in all included studies.

#### 3.3.3 Outcome Measures and Findings

The DMFT index was significantly higher in individuals with PD in two studies [36, 38]. In one study, no significant difference was observed between groups [40]. In two studies, the control group exhibited higher DMFT scores than individuals with PD [39, 45]. In one of these studies [39], the difference was statistically significant. In the other [45], the difference was not significant.

The mean number of teeth with untreated dental caries/dental caries was evaluated in three studies [41, 42, 43]. In two studies, individuals with PD had significantly more untreated dental caries/dental caries than controls [41, 42]. In one study [43], individuals with PD had a significantly lower mean number of teeth with untreated dental caries/dental caries than controls.

The frequency of dental caries was assessed in two studies [37, 44]. In one study [44], individuals with PD exhibited a significantly higher frequency of dental caries than controls.

The DMFS (decayed, missing, and filled surfaces—permanent dentition) index was assessed in one study, showing significantly higher scores in PD patients than controls [38]. The mean number of decayed surfaces was assessed in one study, where controls had significantly more decayed surfaces than PD patients [43].

### 3.4 Risk of Bias Appraisal

The included cross‐sectional studies demonstrated moderate methodological quality. The major concern was the handling of non‐respondents in the selection domain, as no study received a point for this criterion. Regarding exposure appraisal, 60% of the studies adequately evaluated PD. Only two studies implemented strategies to control for confounders [36, 41]. One study [37] did not appropriately report statistical (Supplementary File 3).

### 3.5 Quantitative Synthesis

#### 3.5.1 DMFT Index Meta‐Analysis

**FIGURE 2:**
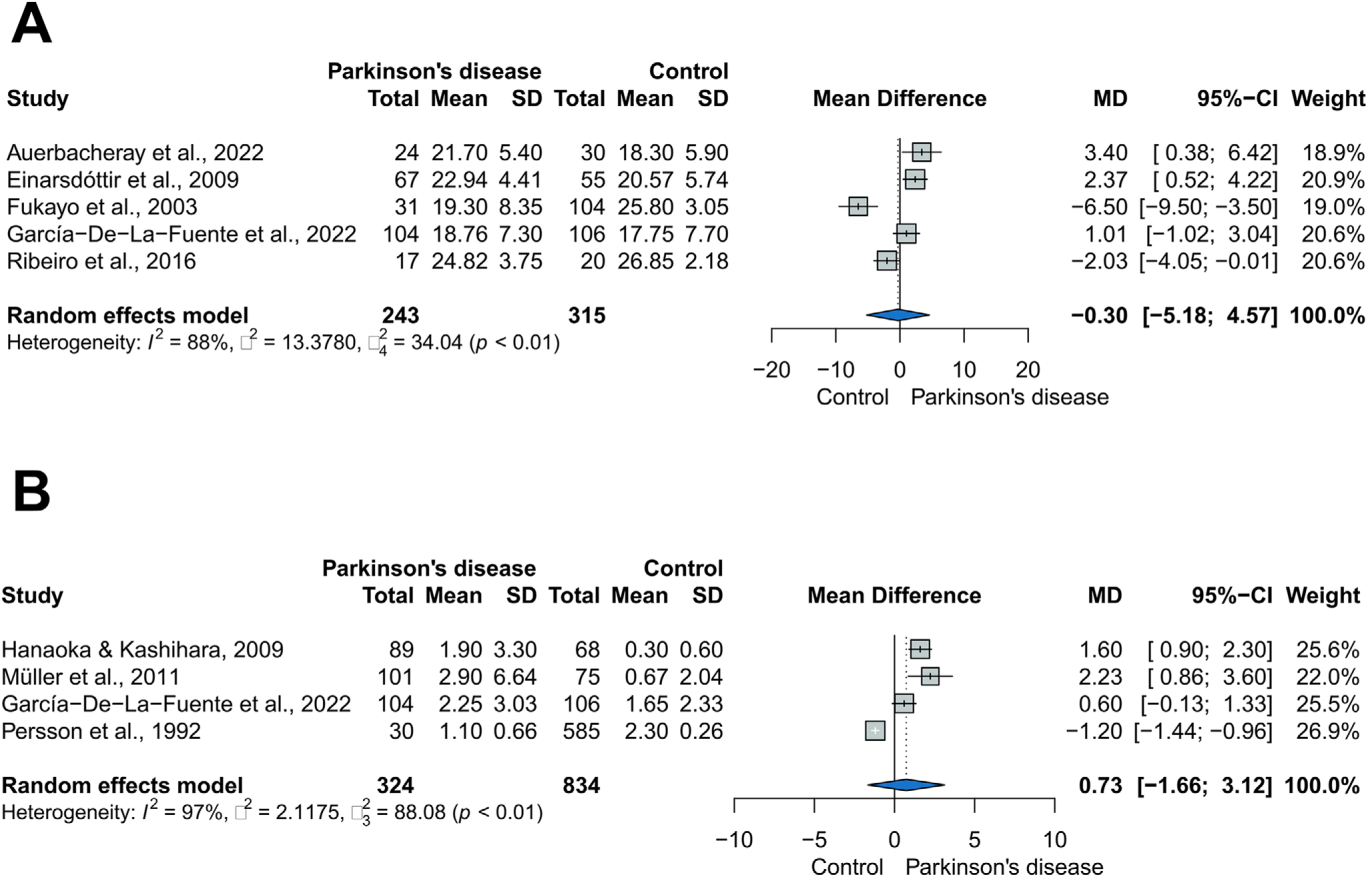
Forest plots displaying the meta‐analyses comparing the **(A)** mean DMFT index and the **(B)** mean number of dental caries/untreated dental caries.

**FIGURE 3:**
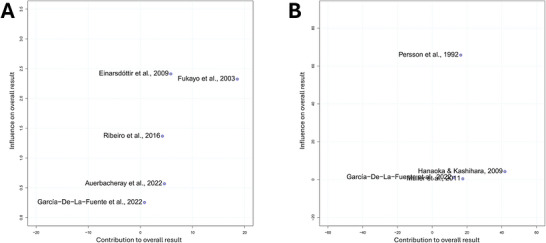
Baujat plot showcasing the influence of the studies in overall heterogeneity in the meta‐analysis of **(A)** DMFT index and **(B)** dental caries/untreated dental caries.

The pooled MD (*k* = 5) between individuals with and without PD was −0.30 (Figure 2A), indicating that PD patients had, on average, 0.30 fewer DMFT units than controls. However, the adjusted 95% CI (−5.18 to 4.57) crossed the null effect size, indicating no statistically significant difference between groups (*t* = −0.17; *p* = 0.870). Heterogeneity was substantial (*I^2^* = 88.2%, *τ* ^2^ = 13.37, and *τ* = 3.65), with a significant *Q*‐statistic (*Q* = 3.73; *df =* 4; *p* < 0.001). The Baujat plot illustrates study influence on heterogeneity (Figure 3A).

#### 3.5.2 Number of Teeth with Untreated Dental Caries Meta‐Analysis

The MD (*k* = 4) was 0.73 (Figure 2B), indicating that PD patients had 0.73 more dental caries/untreated dcarious teeth than controls. However, the adjusted 95% CI (−1.66 to 3.12), crossed the null effect size, indicating no statistically significant difference between groups (*t* = 0.97; *p* = 0.403). Heterogeneity was substantial (*I^2^* = 96.6%, *τ* ^2^ = 2.11, and *τ* = 1.45), with a significant *Q*‐statistic (*Q* = 88.8; *df =* 3; *p* < 0.001). The Baujat plot illustrates study influence on heterogeneity (Figure 3B).

### 3.6 Strength of the Evidence

The GRADE assessment indicated that the strength of the evidence was very low, primarily due to risk of bias, inconsistency, and imprecision across studies (Supplementary File 4).

## 4 Discussion

This systematic review and meta‐analysis did not demonstrate a statistically significant difference in dental caries experience between individuals diagnosed with PD and their age‐matched counterparts without PD. However, this finding should be interpreted as uncertainty in the current evidence base rather than evidence of absence of an association. Both groups exhibited high levels of dental caries experience, underscoring a broader concern regarding aging, longevity, and oral health [47]. Given that participants in primary studies were older adults, with a mean age exceeding 60 years, these findings align with the well‐established association between aging and increased susceptibility to oral diseases [48, 49]. This raises a pertinent question: despite benefiting from increased life expectancy, why do older adults, including those with PD, continue to face unsatisfactory oral health conditions? Both PD and dental caries significantly impact quality of life [24, 25], warranting further investigation into their potential synergistic effects in older adults.

Chronic systemic inflammation is increasingly recognized as a key driver of neurodegeneration in PD [50, 51]; Elevated levels of pro‐inflammatory cytokines have been detected in both the central nervous system and peripheral circulation of individuals with PD, indicating an ongoing inflammatory response that may contribute to disease progression [52]. Importantly, dental caries and periodontitis are also linked to systemic inflammation, suggesting a potential bidirectional relationship between oral health and PD. Oral infections can trigger the release of inflammatory mediators into the bloodstream, exacerbating neuroinflammation,—an effect similarly observed in Alzheimer's disease [53]. Future studies should link biomarkers defining shared and/or overlapping pathophysiological mechanisms.

One plausible explanation for the lack of a significant difference between individuals with and without PD is that the primary studies exclusively included older adults, a population inherently more susceptible to dental caries [48, 49] due to cumulative health burdens over their lifetime [54, 55, 56]. PD typically manifests in later adulthood [4, 9], a stage when individuals have often already experienced various systemic and oral health conditions [57, 58, 59]. Thus, participants in the included studies may have already exhibited not only untreated dental caries, but also tooth loss and restorations, reflecting the cumulative impact of lifetime dental caries experience [60]. This aligns with the established oral health patterns observed in aging populations [61].

Additionally, the DMFT index, i.e., the primary measure used to assess dental caries experience, can be heavily influenced by the “M” component (missing teeth), potentially obscuring the true contribution of the “D” component (decayed teeth) [43, 45]. In older adults, however. missing teeth often reflect cumulative lifetime dental history rather than active caries experience or current caries susceptibility. Consequently, a high DMFT index may be more indicative of a history of extractions than ongoing dental caries activity, further complicating direct comparisons between individuals with and without PD. Future studies should report DMFT components separately and include measures that better capture current disease activity, such as untreated coronal caries, root caries, and incident caries.

As PD progresses, affected individuals often require increased care and support, leading many to reside in care homes [63]. Research indicates that while proper oral hygiene is essential for maintaining quality of life, oral health care in care home settings is often inadequate. Factors such as staff time constraints, lack of dental training, and cognitive or motor impairments in residents contribute to poor oral health outcomes [64]. Regular brushing, ideally twice daily, is critical in reducing plaque accumulation and bacterial load, yet it remains an unmet need in many care facilities. This raises concerns about whether sufficient resources are being allocated to support dental teams in these environments. Given that poor oral hygiene has been associated with cognitive decline and PD, these gaps in care may have broader implications beyond oral health, potentially influencing neurological health in this population [11, 20, 65].

With global life expectancy increasing, the health of older adults has become a cornerstone of the global health agenda, particularly within discussions on longevity and healthy aging [66, 67, 68]. Both PD and oral diseases have been associated with reduced life expectancy and compromised longevity [69, 70, 71], amplifying the challenges of aging. Although current evidence does not demonstrate a widespread or substantial disparity in dental caries prevalence between individuals with and without PD, the potential for a negative synergistic impact of these conditions on quality of life warrants serious consideration by health promoters, policymakers, and stakeholders [12, 13, 65, 72]. Promoting and preserving oral health is paramount in discussions of healthy aging [61, 73] and assumes even greater relevance for older individuals grappling with systemic conditions, such as PD, which significantly impacts their quality of life and well‐being [25].

To mitigate the burden of oral diseases among older adults, particularly those with systemic conditions such as PD, target strategies must be developed and implemented in clinical practice. Given the pivotal role of oral health in healthy ageing [41, 61], dental practitioners play a crucial role in addressing these challenges. A promising solution is home‐based dental care, where mobile dental units and domiciliary visits allow professionals to provide oral health services directly to individuals with PD. By offering routine check‐ups, hygiene instructions, and preventive care in a familiar environment, these services can reduce logistical barriers and improve access to essential dental care [74].

In PD patients, dentists should prioritize preventive strategies and patient education to minimize disease progression and enhance oral health outcomes [75, 76]. While curative treatment remains essential, preventive approach can significantly reduce the need for complex interventions, especially in advanced PD stages, where motor impairments make dental procedures increasingly difficult to perform [20, 77]. One effective intervention is the use of electric toothbrushes, which have demonstrated efficacy in improving biofilm control in individuals with neurodegenerative diseases [36, 78]. Given that brushing proficiency is compromised in PD, electric toothbrushes enhance mechanical plaque removal while reducing the physical effort required for effective hygiene maintenance.

A major limitation of the present study stems from the cross‐sectional design of the included studies, which precludes a longitudinal assessment of the progression of dental caries in PD patients [79]. Consequently, it remains unclear whether Parkinsonian motor symptoms contribute to an increased risk of dental caries over time. Therefore, establishing a causal relationship between PD and dental caries incidence is unfeasible in the current evidence base. Longitudinal studies [80] are required to monitor dental caries development in PD patients over time and compare outcomes with a control group, thereby clarifying whether the nonsignificant difference observed in our meta‐analysis persists or evolves. Another critical drawback is the lack of adequate statistical control for confounders. Nearly all included studies failed to implement multivariate analyses, which may have introduced bias when comparing outcomes between PD and non‐PD groups [81]. This was particularly evident in the risk of bias assessment, highlighting the need for future studies with more robust statistical methodologies. Additionally, we were unable to conduct subgroup analyses to explore sources of heterogeneity, which was notably high in both meta‐analyses [82]. Future research should focus on identifying potential effect modifiers, such as PD severity, disease duration, medication use, salivary dysfunction, dietary factors, socioeconomic status, oral hygiene practices, and access to dental care, to better elucidate the relationship between PD and dental caries risk.

## 5 Conclusion

To date, the available evidence does not demonstrate a statistically significant difference in dental caries experience between older adults with and without PD. However, the certainty of this evidence is very low, and the findings should be interpreted cautiously because they are based exclusively on cross‐sectional studies with substantial heterogeneity and limited control for confounding. The absence of a statistically significant difference should not be interpreted as evidence that PD is unrelated to dental caries susceptibility. Rather, the current literature indicates an important evidence gap and supports the need for longitudinal, confounder‐adjusted studies that evaluate active and incident dental caries outcomes. Prevention remains paramount in managing oral health in this population, particularly as PD progresses and routine dental care may become increasingly burdensome.

## Conflicts of Interest

The authors declare no conflict of interest.

## Funding

This research has not received any specific grants from funding agencies in the public, commercial or non‐profit sectors.

## Supporting information




**Supporting Information**: scd70230‐sup‐0001‐SuppMat.docx

## Data Availability

The data that supports the findings of this study are available in the supplementary material of this article.
