# Supplementary material for: Dental Caries Experience in Older Adults With Parkinson's Disease: A Systematic Review and Meta‐Analysis
Source: Spec Care Dentist. 2026 Aug 10;46(4):e70230. doi: 10.1111/scd.70230 (PMC13454774; doi:10.1111/scd.70230)
Supplement: Supplementary file 1 [file SCD-46-0-s001.docx]

**Supplementary files for Dental caries experience in older adults with Parkinson’s disease: a systematic review and meta-analysis**

**Contents**

[Supplementary File 1: Search strategy employed to identify articles in electronic databases	2](#_Toc189062201)

[Supplementary File 2: Data extracted from cross-sectional studies	3](#_Toc189062202)

[Supplementary File 3: Risk of bias – Newcastle Ottawa Scale (adapted version for cross-sectional studies)	6](#_Toc189062203)

[Supplementary File 4: GRADE (Grading of Recommendations Assessment, Development and Evaluation) analysis: individuals living with SD compared to their coequals in relation to missing teeth	7](#_Toc189062204)

[Supplementary File 5: Scripts used in RStudio to conduct quantitative analysis	8](#_Toc189062205)

# Supplementary File 1: Search strategy employed to identify articles in electronic databases

| **Search strategy** | |
| --- | --- |
| **PubMed, Scopus, Ovid, CINAHL (Ebsco), APA PsycINFO** | “parkinson disease” OR parkinsonism OR “paralysis agitans” OR “lewy body”  AND  “dental caries” OR “dental decay” OR “caries disease” OR “root caries” OR “tooth caries” OR “tooth decay” OR “dental fissure” OR DMFT OR dmft OR DMFS OR dmfs OR ICDAS |
| **Embase, Web of Science** | parkinson disease OR parkinsonism OR paralysis agitans OR lewy body  AND  dental caries OR dental decay OR caries disease OR root caries OR tooth caries OR tooth decay OR dental fissure OR DMFT OR dmft OR DMFS OR dmfs OR ICDAS |

# Supplementary File 2: Data extracted from cross-sectional studies

| **Study (authors name, study design, and country)** | **Sample characteristics** | | **Sample setting** | **Exposure appraisal** | **Outcome appraisal** | **Outcome measures** | |
| --- | --- | --- | --- | --- | --- | --- | --- |
|  | **Study group** | **Control group** |  |  |  | **Study** | **Control** |
| Auerbacheray et al., 2022; cross-sectional; Germany | N= 24 | N= 30 | University dental clinic | NI | Clinical examination | DMFT: 21.7±5.4;  DT: 4±3.3; DFMT (RC) Adjusted: -0.161; DMFT (RC) Sex: -0.01; DMFT (RC) Age: 0.288; DT (RC) Group: -0.540; DT (RC) Sex: 0.033; DT (RC) Age: -0.035 | DMFT: 18.3±5.9; DT: 1±1.9; |
|  | Male: 14 (58.3%) | Male: 11 (33.7%) |  |  |  |  |  |
|  | Female: 10 (41.7%) | Female: 19 (63.3%) |  |  |  |  |  |
|  | Age: 73.0 ±9.0y | Age: 62.5 ±12y |  |  |  |  |  |
| Cicciù et al., 2012; cross-sectional; Italy | N= 45 | N= 45 | Specialized centre | Validated criteria (Hoehn and Yahr scale) | Clinical examination | Untreated Caries (per patient): 3 to 18 (total number: 190/330) | Untreated Caries (per patient): 6 to 14 (total number: 203/418) |
|  | Male: 17 (37.7%) | Male: 35 (77.8%) |  |  |  |  |  |
|  | Female: 28 (62.3%) | Female: 10 (22.2%) |  |  |  |  |  |
|  | Age: 65 to 78y | Age: 65 to 78y |  |  |  |  |  |
| Einarsdóttir et al., 2009; cross-sectional; Iceland | N= 67 | N= 55 | University dental clinic | NI | Clinical examination | DMFT: 22.94±4.41; DMFS: 87.16±28.37 | DMFT: 20.57±5.74; DMFS: 71.95±32.88 |
|  | Male: 39 (58.2%) | Male: 21 (38.2%) |  |  |  |  |  |
|  | Female: 28 (41.8%) | Female: 34 (61.8%) |  |  |  |  |  |
|  | Age: 60 to 70y | Age: 60 to 70y |  |  |  |  |  |
| Fukayo et al., 2003; cross-sectional; Japan | N= 31 | N= 104 | University hospital | Validated criteria (Hoehn and Yahr scale) | Clinical examination | DMFT (Men and SE) - Total: Total :19.3±1.5; 60-69 y (n=12): 14.2±2.1; 70+ (n=19): 22.5±1.6; DMFT – Male: Total (n=17): 20.3±2.0; 60-69 (n=6): 11.5±2.7; 70+: 25.1±1.2; DMFT – Female: Total (n=14): 18±2.2; 60-69 (n=6): 16.8±3.1; 70+(n=8): 18.9±3.2 | DMFT (Men and SE) - Total: Total: 25.8±0.3; 60-69 (n=76): 25.3±0.4; 70+ (n=28): 27.0±0.5; Male: Total (n=61): 25.2±0.5; 60-69 (n=6): 24.9±0.6; 70+: 26.2±0.8; Female: Total (n=43): 26.6±0.4; 60-69 (n=30): 26.0±0.5; 70+ (n=13): 27.9±0.1 |
|  | Male: 17 (54.8%) | Male: 61 (58.65%) |  |  |  |  |  |
|  | Female: 6 (45.2%) | Female: 43 (41.35%) |  |  |  |  |  |
|  | Age: over 60y | Age: over 60y |  |  |  |  |  |
| García-De-La-Fuente et al., 2022; cross-sectional; Spain | N= 104 | N= 106 | University dental clinic | NI | Clinical examination | DMFT: 18.76±7.3; Dental caries: 2.25±3.03 | DMFT: 17.75 ±7.7; Dental caries: 1.65±2.33 |
|  | Male: 66 (63.5%) | Male: 37 (34.9%) |  |  |  |  |  |
|  | Female: 38 (36.5%) | Female: 69 (65.1%) |  |  |  |  |  |
|  | Age: 66±9.3y | Age: 59.2 ±14.1y |  |  |  |  |  |
| Hanaoka & Kashihara, 2009; cross-sectional; Japan | N= 89 | N= 68 | NI | Validated criteria (Hoehn and Yahr scale) | Clinical examination | Untreated caries (%): 53.5; Untreated caries: 1.9±3.3 | Untreated caries (%): 8.1; Untreated caries: 0.1±0.6 |
|  | Male: 38 (42.7%) | Male: 26 (38.3%) |  |  |  |  |  |
|  | Female: 51 (57.3%) | Female: 42 (61.7%) |  |  |  |  |  |
|  | Age: 72.1±5.5y | Age: 69±5.8y |  |  |  |  |  |
| Müller et al., 2011; cross-sectional; Germany | N= 101 | N= 75 | Hospital (Neurology department) | Validated criteria (Hoehn and Yahr scale) | Clinical examination | Decayed teeth: 2.90±6.64; Decayed teeth (male): 4.20±8.39; Decayed teeth (female): 1.35±3.01 | Decayed teeth: 0.67±2.04; Decayed teeth (male): 0.86±2.6; Decayed teeth (female): 0.50±1.40 |
|  | Male: 55 (54.4%) | Male: 35 (46.6%) |  |  |  |  |  |
|  | Female: 46 (45.6%) | Female: 40 53.4%) |  |  |  |  |  |
|  | Age: 66.2±10.5y | Age: 71±10.53y |  |  |  |  |  |
| Persson et al., 1992; cross-sectional; Sweden | N= 30 | N= 585 | Hospital (Geriatric department) | Validated criteria | Clinical examination | Decayed teeth: 1.1±0.66; Decayed surfaces: 3.4±2.0 | Decayed teeth: 2.3±0.26; Decayed surfaces: 7.0±1.14 |
|  | Male: 17 (56.6%) | Male: NI |  |  |  |  |  |
|  | Female: 13 (43.4%) | Female: NI |  |  |  |  |  |
|  | Age: 73±7.3y | Age: NI |  |  |  |  |  |
| van Stiphout et al., 2018; cross-sectional; the Netherlands | N= 65 | N= 65 | University neurology clinic | Validated criteria (Hoehn and Yahr scale) | Clinical examination | Teeth with caries (n): 74/74 | Teeth with caries (n): 12/74 |
|  | Male: NI | Male: NI |  |  |  |  |  |
|  | Female: NI | Female: NI |  |  |  |  |  |
|  | Age: NI | Age: NI |  |  |  |  |  |
| Ribeiro et al., 2016; cross-sectional; Brazil | N= 17 | N= 20 | Specialized centre | NI | Clinical examination | DMFT: 24.82±3.75 | DMFT: 26.85±2.18 |
|  | Male: 9 (53%) | Male: 10 (50%) |  |  |  |  |  |
|  | Female: 8 (47%) | Female: 10 (50%) |  |  |  |  |  |
|  | Male: NI | Male: NI |  |  |  |  |  |
|  | Female: NI | Female: NI |  |  |  |  |  |
|  | Age: 69.41±4.65y | Age: 72.0±5.69y |  |  |  |  |  |

Note: NI, not informed; DMFT, decayed, missing, and filled teeth; DT, decayed teeth; RC, regression coefficient; SE, standard error;

# Supplementary File 3: Risk of bias – Newcastle Ottawa Scale (adapted version for cross-sectional studies)

| Study/reference | Selection^*^ | | | | Comparability^**^ | Outcome^***^ | |
| --- | --- | --- | --- | --- | --- | --- | --- |
|  | Representativeness of the sample^1^ | Sample size^2^ | Non-respondents^3^ | Ascertainment of the exposure^4^ | The subjects in different outcome groups are comparable, based on the study design or analysis. Confounding factors are controlled.^5^ | Assessment of outcome^6^ | Statistical test^7^ |
| Auerbacheray et al, 2022 | ✯ | ✯ |  |  | ✯✯ | ✯✯ | ✯ |
| Cicciù et al, 2012 | ✯ | ✯ |  | ✯✯ |  | ✯✯ |  |
| Einarsdóttir et al, 2009 | ✯ | ✯ |  |  |  | ✯✯ | ✯ |
| Fukayo et al, 2003 | ✯ | ✯ |  | ✯✯ |  | ✯✯ | ✯ |
| García-De-La-Fuente et al, 2022 | ✯ | ✯ |  |  |  | ✯✯ | ✯ |
| Hanaoka & Kashihara, 2009 | ✯ | ✯ |  | ✯✯ | ✯✯ | ✯✯ | ✯ |
| Müller et al., 2011 | ✯ | ✯ |  | ✯✯ |  | ✯✯ | ✯ |
| Persson et al., 1992 | ✯ | ✯ |  | ✯ |  | ✯✯ | ✯ |
| van Stiphout et al, 2018 | ✯ | ✯ |  | ✯✯ |  | ✯✯ | ✯ |
| Ribeiro et al., 2016 | ✯ | ✯ |  |  |  | ✯✯ | ✯ |

^*^a maximum of 1 point for the first three items and a maximum of 2 for the last item, being the total 5 points; ^**^a maximum of 2 points for each item; ^***^a maximum of 2 points for the first item and a maximum of 1 for the last item, being the total 3 points.

✯1 Point.

^1^a) Truly representative of the average in the target population ✯; b) Somewhat representative of the average in the target population ✯; c) Selected group of users; d) No description of the sampling strategy.

^2^a) Justified and satisfactory ✯; b) Not justified.

^3^a) Comparability between respondents’ and non-respondents’ characteristics is established, and the response rate is satisfactory ✯; b) The response rate is unsatisfactory, or the comparability between respondents and non-respondents is unsatisfactory; c) No description of the response rate or the characteristics of the responders and the non-responders.

^4^a) Validated measurement tool ✯✯; b) Non-validated measurement tool, but the tool is available or described ✯; c) No description of the measurement tool.

^5^a) The study controls for the most important factor ✯; b) The study control for any additional factor ✯.

^6^a) Independent blind assessment ✯✯; b) Record linkage ✯✯; c) Self report ✯; d) No description;

**Supplementary File 4: GRADE (Grading of Recommendations Assessment, Development and Evaluation) analysis: individuals living with SD compared to their coequals in relation to missing teeth**

| **Certainty assessment** | | | | | | | **Number of patients** | | **Effect** | | Certainty |
| --- | --- | --- | --- | --- | --- | --- | --- | --- | --- | --- | --- |
| Number of studies | Study design | Risk of bias | Inconsistency | Indirectness | Imprecision | Other considerations | **With Parkinson’s disease (95% CI)** | **Without Parkinson’s disease (95% CI)** | **Relative** | **Absolute** |  |
| ***DMFT Index*** | | | | | | | | | | | |
| 5 | Non-randomized studies | Serious^a^ | Serious^b^ | Not serious | Serious^c^ | strong association; all plausible residual confounding would reduce the demonstrated effect | 243 | 315 | - | MD **0.3 lower** (5.18 lower to 4.57 higher) | ⨁◯◯◯ Very Low^a,b,c^ |
| *Dental caries/untreated dental caries* | | | | | | | | | | | |
| 4 | Non-randomized studies | Serious^a^ | Serious^b^ | Not serious | Serious^c^ | strong association; all plausible residual confounding would reduce the demonstrated effect | 324 | 834 | - | MD **0.73 higher** (1.66 lower to 3.12 higher) | ⨁◯◯◯ Very Low^a,b,c^ |

Explanations: a. The evidence was downgraded due to methodological limitations of the included studies, particularly limited control for confounding and incomplete reporting of non-respondents. b. The evidence was downgraded due to high heterogeneity, indicating substantial variation in study results that could not be fully explained. c. The evidence was downgraded due to imprecision, because the confidence intervals were wide and crossed the null effect, including both potentially meaningful harm and benefit. No upgrading criteria were applied.

# Supplementary File 5: Scripts used in RStudio to conduct quantitative analysis

```
##################Parkinson Disease - DMFT
library(meta)
library(openxlsx)

DMFT = metacont(pd_n, pd_mean, pd_sd, c_n, c_mean, c_sd,
                studlab = paste(study), data = DMFT,
                fixed = F, sm = "MD", hakn = T, prediction = T,
                method.tau = "REML")

print(DMFT)

forest(DMFT, label.left = "Control", label.right = "Parkinson's disease",
       lab.e = "Parkinson's disease", digits.sd = 2, col.diamond = "dodgerblue3",
       col.diamond.lines = "black",
       col.square = "azure3", xlim = c(-25,25), print.tau2 = T, print.I2.ci = F,
       print.Q = T, print.pval.Q = T, squaresize = 0.7,
       col.square.lines = "black", prediction = F)

baujat(DMFT, col="blue", cex = 1.5, xlim = c(-20, 20), ylim = c(0.0, 3.5),
       cex.studlab = 1.8, col.grid = "lightblue")


##################Parkinson Disease - Dental Caries
DC = metacont(pd_n, pd_mean, pd_sd, c_n, c_mean, c_sd,
              studlab = paste(study), data = Dental_caries,
              fixed = F, sm = "MD", hakn = T, prediction = T,
              method.tau = "REML")

print(DC)

forest(DC, label.left = "Control", label.right = "Parkinson's disease",
       lab.e = "Parkinson's disease", digits.sd = 2, col.diamond = "dodgerblue3",
       col.diamond.lines = "black",
       col.square = "azure3", xlim = c(-10,10), print.tau2 = T, print.I2.ci = F,
       print.Q = T, print.pval.Q = T, squaresize = 0.7,
       col.square.lines = "black", prediction = F)


baujat(DC, col="blue", cex = 1.5, xlim = c(-60, 60), ylim = c(-20, 90),
       cex.studlab = 1.8, col.grid = "lightblue")
```
